# Buildings Contemplated and in Progress

**Published:** 1914-05-16

**Authors:** 


### Buildings Contemplated and in Progress.

Ashington.—The medical officers of the Morpeth Rural District Council, the Ashington Urban District Council, and the Newbiggin Urban District Council have been asked to submit a joint report in regard to the provision of an infectious diseases hospital for the three areas.

Ashton-under-Lyne.—The proposed extensions at the Ashton District Infirmary will provide accommodation for an additional fifty or sixty beds, a new wing with an additional operating theatre, a Röntgen-ray department, and an eye department. A site will be selected adjoining the Kershaw Hospital. The scheme will involve an expenditure of about £25,000.

Bedwellty.—The Monmouthshire Health and Housing Committee have agreed to the principle of grouping the seven urban districts in the west of the county for the provision of isolation hospitals. It is contemplated to erect one hospital at or near Pontllanfraith and another on or near Bedwellty Common.

Brentford.—The Middlesex County Council have appointed a visiting committee to consider the provision of an additional mental hospital.

Bromley.—The Bromley and Beckenham Joint Hospital Board invites tenders for certain alterations and additions to the isolation hospital at Bromley Common.

Denbigh.—The Welsh Insurance Commissioners have approved the acquisition of the Plas Langwyfan estate, near Denbigh, as a site for a sanatorium, to be built and managed by the King Edward Memorial Association.

Dublin.—Tenders are invited for the erection of an isolation hospital, etc., at Portraine Asylum. Mr. D. Morris is the architect. Quantities may be obtained of Messrs. Morris and Kavanagh, 68 Harcourt Street, Dublin.

Ealing.—The Local Government Board have approved the plans for the proposed dispensary at Ealing, and the County Council will obtain tenders for the work.

Exeter.—The Governors of the Royal Devon and Exeter Hospital propose to build a new wing for nurses. The plans have been prepared by Mr. E. H. Harbottle, the honorary surveyor.

Guildford.—The Guildford, Godalming, and Woking Joint Hospital Board have instructed Mr. J. Herbert Norris, architect and surveyor, Godalming, to report upon and to submit estimates for alterations and additions to the hospital.

Hastings.—The work of preparing for the King Edward Memorial Hospital has been begun, and the governors have authorised the committee to spend a sum not exceeding £50,000 on the building.

Leeds.—It is proposed to carry out various extensions and alterations at Leeds Hospital, at a cost of £8,471.

Mexborough.—Tenders are invited for the erection of a new ward and enlargement of the kitchen block at the Montagu Hospital, Mexborough. Messrs. Geo. Moxon and Son, Central Chambers, 26 Church Street, Barnsley, are the architects, from whom quantities may be obtained.

Sheffield.—The Guardians have decided to borrow £6,260 for alterations and additions to the nurses' home.

Shelf.—The new Halifax Corporation Sanatorium, built at Shelf at a cost of £6,700, has been formally opened.

### West Kent General Hospital, Maidstone.

In Sir Henry Burdett's Report on this hospital, published last week, the following omission occurred :—On page 153, first column, line 10, under the head of "Undesirable Fees," after the words "100 beds" the following words should have appeared : "of which forty-five on an average are occupied." The full sentence should read as follows :—"These fees ought not to have been levied, seeing that the size of the hospital, which contains less than 100 beds, of which forty-five on an average are occupied, is not sufficient to enable the nurses to be properly trained or certificated at the end of three years' work at the institution."

